# Interactions of PARP1 Inhibitors with PARP1-Nucleosome Complexes

**DOI:** 10.3390/cells11213343

**Published:** 2022-10-23

**Authors:** Natalya Maluchenko, Darya Koshkina, Anna Korovina, Vasily Studitsky, Alexey Feofanov

**Affiliations:** 1 Biology Faculty, Lomonosov Moscow State University, 119234 Moscow, Russia; 2 Institute of Gene Biology RAS, 34/5 Vavilov Str., 119334 Moscow, Russia; 3 Fox Chase Cancer Center, Philadelphia, PA 19111, USA

**Keywords:** poly(ADP-ribose-)polymerase-1, PARP1 inhibitors, talazoparib, olaparib, veliparib, nucleosome, chromatin

## Abstract

Inhibitors (PARPi) of poly(ADP-ribose-)polymerase-1 (PARP1) are used in antitumor therapy; their cytotoxicity correlates with the efficiency of PARP1 trapping in cell chromatin. Previous studies have demonstrated the PARPi-induced trapping of PARP1 on DNA, although details of the mechanism remain controversial. Here, the interactions of PARP1-nucleosome complexes with PARPi, olaparib (Ola), talazoparib (Tala), and veliparib (Veli) were studied. PARPi trap PARP1 on nucleosomes without affecting the structure of PARP1-nucleosome complexes. The efficiency of PARP1 trapping on nucleosomes increases in the order of Tala>Ola>>Veli, recapitulating the relative trapping efficiencies of PARPi in cells, but different from the relative potency of PARPi to inhibit the catalytic activity of PARP1. The efficiency of PARP1 trapping on nucleosomes correlates with the level of inhibition of auto-PARylation, which otherwise promotes the dissociation of PARP1-nucleosome complexes. The trapping efficiencies of Tala and Ola (but not Veli) are additionally modulated by the enhanced PARP1 binding to nucleosomes. The dissociation of PARP1-nucleosome complexes occurs without a loss of histones and leads to the restoration of the intact structure of nucleosomal DNA. The data suggest that the chromatin structure can considerably affect the efficiency of the PARPi action.

## 1. Introduction

Poly(ADP-ribose)polymerase 1 (PARP1) is a nuclear protein which is involved in DNA repair, replication and transcription, cell cycle regulation, and programmed cell death [1,2,3]. The participation of PARP1 in a variety of critical cellular processes is determined to a large extent by its DNA-binding and catalytic activity [3,4].

The DNA-binding domains of PARP1 are represented by three zinc fingers that are able to recognize various DNA structures in chromatin, including DNA breaks and non-canonical forms of DNA. DNA binding is accompanied by conformational changes and the activation of the catalytic activity of PARP1. Activated PARP1 catalyzes the transfer of adenosine diphosphate-ribose residue from NAD+ to target proteins that results in the formation of up to 90% of all polyADP-ribose chains (PAR) in a cell [5,6,7]. In turn, polyADP-ribosylation (PARylation) initiates a variety of PAR-mediated cellular processes [3]. The impaired PARP1 metabolism is associated with the development of tumors, and with cardiovascular and neurodegenerative diseases [4,8].

Some inhibitors of PARP1 (PARPi) are approved for antitumor therapy and are being evaluated for the treatment of other metabolic diseases [4,9,10]. Known PARPi are nicotinamide mimetics that bind to the catalytic domain of PARP1 and interfere with NAD+ binding. The cytotoxicity of PARPi is the result of the inhibition of PARylation and the trapping of PARP1 complexes in chromatin; these two mechanisms can complement and reinforce each other [11,12]. The inhibition of PARylation leads to synthetic lethality in cells which are deficient in some DNA double break repair enzymes [13]. The trapping of PARP1-DNA complexes at the chromatin creates obstacles to the normal course of nuclear processes, such as transcription or the passage of a replication fork, which ultimately leads to cell death. PARP1 trapping was detected when the cells were treated either with PARPi alone [12,14,15,16] or with a combination of PARPi and DNA alkylating agents such as methyl methanesulfonate or temozolomide [11,17,18]. The trapping efficiency, rather than the ability to inhibit the catalytic activity of PARP1, correlates with the cytotoxicity of PARPi [12,17,19].

The mechanism of the trapping of PARP1 complexes is the subject of active research [12,14,15,16,17,18,19,20,21]. It has been suggested that the PARPi-dependent PARP1 trapping at sites of DNA damage is explained by the inhibition of the auto-PARylation of PARP1, which normally promotes the dissociation of PARP1-DNA complexes [12,14,15,16]. Alternatively, the reverse allostery hypothesis suggests that PARP1 trapping is defined by an increase in the affinity of PARP1 to DNA resulting from the PARPi-induced conformational changes in the protein [11,17,18], which propagate from the catalytic domain to the helical domain, the so called WGR domain and the DNA binding domain [22]. The first possibility is supported mainly by the results of experiments on DNA in vitro, while the reverse allostery hypothesis is based on the studies of chromatin isolated from cells treated with PARPi. Studies of PARP1 trapping in complexes with DNA (short oligonucleotides) in vitro produced controversial results, either confirming [17,18,21] or refuting [12,15,16] the possibility that PARP1 inhibition is modulated by the additional mechanism of trapping. An extended study of PARPi revealed that some of them enhance the retention of PARP1 on DNA, while others, on the contrary, facilitate its release [20].The interaction of PARP1 with DNA in the presence of PARPi was supposed to occur by a two-stage mechanism, which includes the stage of catalytic inhibition by PARPi, followed by an allosteric modulation of the interactions between PARP1 and DNA [23].

It should be noted that some additional factors can modulate the activity of PARPiin cells. For example, histone PARylation factor 1 (HPF1) significantly increases the affinity of some PARPi for PARP1 [24]. It cannot be excluded that some topological features of DNA in chromatin modulate DNA interactions with PARP1 and PARPi.

**Figure 1:**
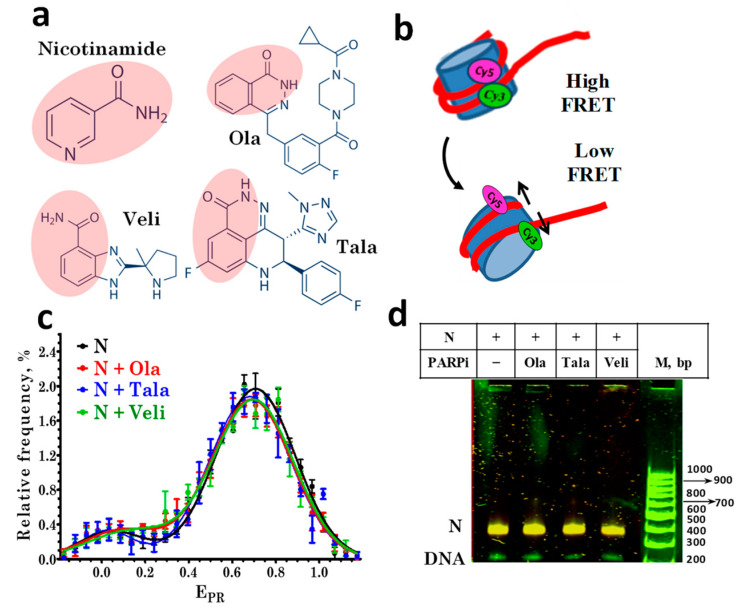
PARP1 inhibitors do not affect the nucleosome structure. (**a**) Structures of nicotinamide and the studied PARPi: olaparib (Ola), talazoparib (Tala), and veliparib (Veli). Pink shading indicates the nicotinamide pharmacophore in the PARPi structures. (**b**) Changes in FRET efficiency induced by alterations in the nucleosome structure. In intact nucleosomes, FRET efficiency is high due to the proximity of Cy3 and Cy5 localized on adjacent DNA gyres. If the nucleosome structure changes are accompanied by an increase in the distance between labels, the efficiency of FRET decreases. (**c**) E_PR_ profiles of nucleosomes in the absence and presence of 10 µM PARPi. N-nucleosomes. E_PR_ profiles are presented as the mean ± standard error of the mean (*n* = 3). (**d**) EMSA data for nucleosomes (N) in the absence and presence of 10 μM PARPi: analysis of FRET in gel. The color scheme used to distinguish different FRET efficiencies for nucleosomes in gel was as follows. FRET efficiency decreases in the order: orange> yellow > green color. The same yellow color of the nucleosome bands indicates the absence of structural changes in nucleosomes caused by PARPi.

In this work, the interactions of PARPi with PARP1-nucleosome complexes were studied using mononucleosomes as a model system for the analysis of the intermolecular interactions in chromatin. To study the effect of PARP1 and PARPi on the structure of nucleosomes, a pair of fluorescent labels was introduced into neighboring gyres of nucleosomal DNA, and the structural changes were monitored by the measuring efficiency of a Förster resonance energy transfer (FRET) between these labels at the level of single supramolecular complexes. The effects of three PARPi (olaparib (Ola), talazoparib (Tala), and veliparib (Veli), Figure 1a), which differ in the efficiency of PARP1 trapping in complexes with chromatin in cells [14,17,20], on the structure of nucleosomes and PARP1-nucleosome complexes were studied. The PARPi-induced trapping of PARP1 in complexes with nucleosomes was demonstrated, and the differences between Ola, Tala, and Veli in the efficiency of PARP1 trapping were characterized.

## 2. Materials and Methods

Ola, Tala, and Veli (Selleck, Houston, TX, USA) were dissolved in dimethylsulfoxide at a concentration of 10 mM and stored at −20 °C.

Fluorescently labeled 167 bp DNA templates with the nucleosome-positioning sequence 603-42A were obtained by a polymerase chain reaction (PCR) using the following primers labeled with Cy3 and Cy5 fluorophores (Lumiprobe, Cockeysville, MD, USA):

direct—5′-CAAgCgACACCggCACTgggCCCggTTCgCgC[Cy3-dT]CCCgCCTTCCg TgTgTTgTCgTCTCTCgggCgT-3′;

reverse—5′-ACCCCAgggACTTgAAgTAATAAggACggAgggCCTCTTTCAACATC gATgCACgg[Cy5-dT]ggTTAg- -3′.

The use of this pair of primers ensured the introduction of Cy3 and Cy5 into the DNA template at positions 13 and 91 bp from the beginning of the nucleosome-positioning sequence, respectively.

To assemble nucleosomes on DNA templates, chromatin without H1 histone was used as a donor of the core histones. It was isolated from chicken erythrocytes, as described previously [25]. Nucleosomes were assembled by stepwise dialysis against a decreasing NaCl concentration at 4 °C, according to the protocol described in [25]. Nucleosomes were purified from excess donor chromatin and nonspecific reaction products using preparative electrophoresis in 4% polyacrylamide gel (PAGE) in 10 mM of HEPES-NaOH buffer (Sigma-Aldrich, Merck, Germany) pH 8.0; 0.2 mM EDTA. Pre-electrophoresis (150 V, 1.5 h, 4 °C) was performed before applying the samples to the gel. A fluorescent analysis of nucleosomes in gel was performed using an Amersham Typhoon RGB imager (GE Healthcare Bio-Sciences AB, Uppsala, Sweden). Based on the obtained images, the target PAGE bands with nucleosomes were cut out and crushed. Nucleosomes were eluted from the gel with a buffer containing 10 mM of HEPES-NaOH (pH 8.0), 0.2 mM of EDTA, 200 μg/mL of bovine serum albumin and stored at +4 °C. Recombinant human PARP1 was expressed in *E. coli* cells, purified as described in [26], and stored at −80 °C.

All the studies were performed in a solution containing 150 mM KCl, 20 mM Tris-HCl (pH 7.5), 5 mM MgCl_2_, and 1 mM β-mercaptoethanol. The samples were prepared by incubating nucleosomes (1–2 nM) with PARP1 (15 or 50 nM) for 30 min at +25 °C. For the poly-ADP-ribosylation reaction, 100 μM of NAD+ (Merck, Germany) was added in 15 min after mixing nucleosome with PARP1, and the reaction mixture was further incubated for 35 min. PARPi (0.1–10 µM) was added to PARP1 15 min before mixing with the nucleosomes. The concentration of DMSO in the mixtures with PARPi was less than 1%. It has been shown that the presence of 1% of DMSO or less does not affect the structure of the nucleosomes.

The FRET-based single-particle microscopy measurements (spFRET microscopy) were performed using the LSM710-Confocor 3 confocal microscope (Carl Zeiss, Aalen, Germany) in silicone wells fixed on a cover glass. A water-immersion 40× C-Apochromat objective (numerical aperture 1.2) was used. The Cy3 donor was excited at the 514.5 nm wavelength (1 μW at the sample), and the fluorescence intensities of the Cy3 donor and Cy5 acceptor were recorded in the 530–635 nm and 635–800 nm ranges, respectively. Signals from single freely diffusing nucleosomes and their complexes were recorded from nucleosome solutions diluted to ~1 nM, providing no more than one nucleosome in the focus of the laser beam at any time. The signal integration time was 3 ms, and the duration of each of the 2–3 successive measurements was 10 min. The fluorescence intensities of Cy3 and Cy5 (I_3_ and I_5_) measured from single nucleosomes were corrected for the background and recalculated to the proximity ratio coefficient (E_PR_) using the equation

E_PR_ = (I_5_ − 0.19 × I_3_)/(I_5_ + 0.81 × I_3_),

where the coefficients 0.19 and 0.81 accounted for the partial overlap of the fluorescence spectra of Cy3 and Cy5 in the region of 635–800 nm [27]. The E_PR_ coefficient is an analogue of the FRET efficiency without corrections for fluorophore quantum yields and differences in instrument sensitivity in the Cy3 and Cy5 fluorescence emission ranges. The calculation results were presented as histograms of the distribution of nucleosomes and their complexes with PARP1 according to the E_PR_ value (E_PR_ profiles). The data were obtained from several independent experiments, and the sample sizes were at least 3000 nucleosomes. The E_PR_ profiles were described as a superposition of several normal distributions (Gaussian bands) corresponding to different structural states of nucleosomes. The proportion of nucleosomes in each of these states was determined as the ratio of the area under the corresponding Gaussian band to the total area under the E_PR_ profile.

The samples for electrophoretic mobility shift assays (EMSA) were prepared as for spFRET microscopy, but the concentration of nucleosomes was 3 nM. Electrophoresis in 4% gel subjected to pre-electrophoresis was carried out using a 0.2× TBE buffer containing 3.6 mM of Tris (pH 7.5), 3.6 mM of boric acid, 0.08 mM of EDTA at 100 V and +4 °C for 50 min. Fluorescent analysis of the gels was performed using the Amersham Typhoon RGB laser scanner (GE Healthcare Bio-Sciences Corp., Marlborough, MA, USA) at the 532 nm excitation wavelength. Fluorescent images were recorded at 580 nm (Cy3 emission) and 670 nm (Cy5 emission). To visualize FRET in the gel, two fluorescent images were merged using the ImageJ program (National Institutes of Health, Bethesda, MD, USA), assigning green and red colors to the Cy3 and Cy5 images, respectively. This algorithm made it possible to qualitatively evaluate the FRET efficiency in each of the electrophoresis bands by color. The FRET efficiency decreases in the following order: green < yellow < orange color.

## 3. Results

### 3.1. PARP1 Inhibitors Do Not Affect the Nucleosome Structure

The structural features of the assembled fluorescently labeled nucleosomes were characterized by spFRET microscopy in the presence of various PARPi (Figure 1a), based on a recording of the fluorescence intensities of Cy3 and Cy5 labels in single nucleosomes (Figure 1b) freely diffusing through the focus of the laser beam. According to the analysis of the E_PR_ profiles, nucleosomes are characterized by the presence of two subpopulations that differ in FRET efficiency: the main subpopulation with a maximum of E_PR_ = 0.71, and a minor subpopulation with a maximum of E_PR_ = 0.03 (Figure 1c).

The main subpopulation is formed by nucleosomes with an intact structure, and the minor subpopulation is formed by free DNA and/or nucleosomes, in which the distance between DNA gyres increased in the region of the label location due to the so-called nucleosome “breathing”, i.e., spontaneous reversible unfolding of DNA from the histone octamer near the DNA entrance to the nucleosome [28].

The incubation of nucleosomes with Ola, Tala, or Veli at a concentration of 10 μM did not cause significant changes in the E_PR_ profile of nucleosomes (Figure 1c), their electrophoretic mobility or FRET efficiency, measured from the nucleosomes in the gel (Figure 1d). No changes were found in the E_PR_ profile of the nucleosomes up to the 100 μM concentration of PARPi (data not shown). Thus, the studied PARPi do not affect the structure of nucleosomes in a wide range of concentrations.

### 3.2. Interaction of PARPi with Nucleosome-PARP1 Complexes

**Figure 2:**
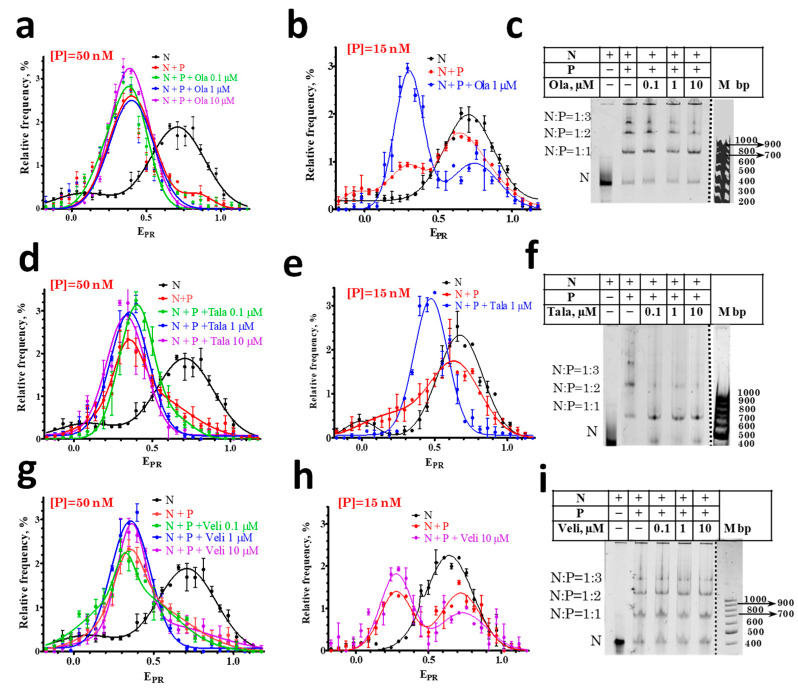
Effect of PARPi on the interaction of PARP1 with nucleosomes. (**a**,**b**,**d**,**e**,**g**,**h**) E_PR_ profiles of nucleosomes (N) and nucleosome-PARP1 complexes (N+P) at different indicated concentrations of PARPi. Concentration of PARP1 ([P]) was 50 nM (**a**,**d**,**g**) or 15 nM (**b**,**e**,**h**). E_PR_ profiles are presented as mean ± standard error of the mean (*n* = 3). (**c**,**f**,**i**) EMSA data for nucleosomes and nucleosome-PARP1 complexes at the 50 nM concentration of PARP1 and different concentrations of PARPi. The stoichiometry of nucleosome-PARP1 complexes is indicated on the left. Some discrepancy between spFRET data in solution and in the gel, namely, appearance of small fraction of free nucleosomes (**c**,**f**,**i**) are most likely explained by a decreased stability of the complexes during electrophoresis.

In agreement with the previously published data [29], PARP1 forms three types of complexes with the nucleosomes, which differ in the number of enzyme molecules associated with one nucleosome: 1:1, 2:1, and 3:1 (Figure 2c,f,i). The formation of complexes changes the DNA folding in the nucleosome [29], which is detected by spFRET microscopy as a decrease in the E_PR_ values for the main subpopulation of the nucleosomes from 0.7 to 0.4 (Figure 2a,d,g). A decrease in the E_PR_ value indicates an increase in the distance between the nucleosomal DNA gyres near the sites of Cy3 and Cy5 attachment. Two PARP1 molecules are most probably bound to the ends of nucleosomal DNA. The third PARP1 molecule is supposed to be bound to a core region of a nucleosome and seems to be mainly responsible for the structural rearrangement of a nucleosome [29]. As we have demonstrated earlier, the dissociation of PARP1 from the complex leads to the recovery of the intact nucleosome structure [30].

spFRET microscopy studies revealed that the E_PR_ profile of nucleosome-PARP1 complexes does not change when the concentration of PARP1 is 50 nM and different PARPi are added at concentrations of 0.1–10 μM (Figure 2a,d,g). At this concentration of PARP1, almost all of the nucleosomes are in the complexes, and PARPi do not affect the conformation of nucleosomal DNA within nucleosome-PARP1 complexes.

Additional experiments were performed at the 15 nM PARP1 concentration. According to the E_PR_ profile, only a fraction of the nucleosomes form complexes with PARP1 at this concentration: the E_PR_ profile is a superposition of the peak at 0.71 that corresponds to free nucleosomes and the peak in the region of 0.3–0.4 that corresponds to PARP1-nucleosome complexes (Figure 2b,e,h). The addition of Ola or Tala causes an increase in the amplitude of the peak corresponding to the PARP1-nucleosome complexes and a decrease in the amplitude of the peak of the free nucleosomes, that can be explained by the PARPi-induced shift in the equilibrium between the free and bound nucleosomes towards the formation of PARP1-nucleosome complexes (Figure 2b,e). Tala induces a more pronounced effect than Ola (Figure 2b,e). EMSA confirms that the addition of 1 µM of Ola or Tala enhances the formation of the PARP1-nucleosome complexes (Figure 2c,f). In contrast, Veli does not affect the equilibrium between the free and bound nucleosomes, even at the 10 µM concentration (Figure 2h).

### 3.3. Effect of PARPi on the Interaction of NAD+ with Nucleosome-PARP1 Complexes

**Figure 3:**
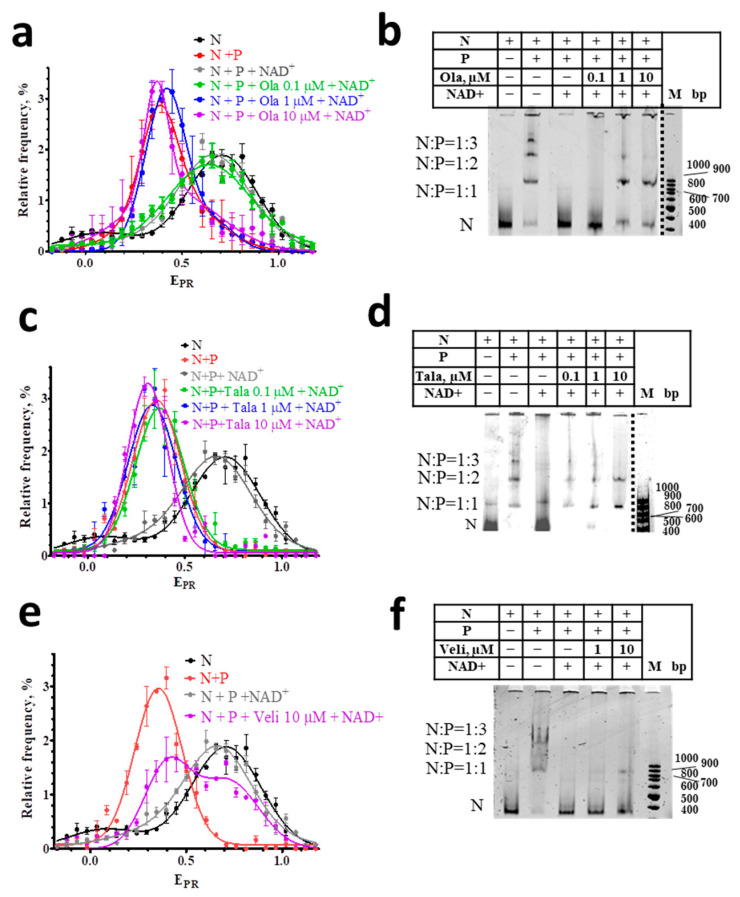
Effect of NAD+ on nucleosome-PARP1-PARPi complexes. (**a**,**c**,**e**) E_PR_ profiles of nucleosomes (N) and nucleosome-PARP1 complexes (N+P) treated with NAD+ (100 µM) in the presence of different concentrations of Ola (**a**), Tala (**c**), and Veli (**e**). Concentration of PARP1 was 50 nM. E_PR_ profiles are presented as mean ± standard error of the mean (*n* = 3). (**b**,**d**,**f**) EMSA data for nucleosomes and their complexes with PARP1 (50 nM) treated with NAD+ (100 µM) and different concentrations of Ola (**b**), Tala (**d**), and Veli (**f**).

It is known that the binding of PARP1 to DNA activates the enzyme, and initiates the NAD+-induced PARylation of nearby proteins and PARP1 itself [31,32,33,34,35]. The PARylation of PARP1 leads to the dissociation of the PARP1-nucleosome complexes and the almost complete restoration of the native structure of the released nucleosomes [29], despite the possible PARylation of histones [33], which strongly depends on the presence of HPF1 (histone PARylation factor 1) [24,36]. The restoration of the intact structure of nucleosomes is confirmed by the close similarity of the E_PR_ profiles of free nucleosomes before the interaction with PARP1 and after their release from the complexes in the presence of NAD+ (Figure 3a,c,e).

spFRET analysis also shows that the interaction of PARPi with nucleosome-PARP1 complexes significantly compromises the response of these complexes to NAD+. In the presence of 10 μM of Ola or Tala, NAD^+^ does not cause the changes in the E_PR_ profiles of nucleosome-PARP1 complexes, which are observed in the absence of PARPi (Figure 3a,c). This result indicates that Ola and Tala effectively inhibit the (poly-ADP) ribosylation reaction and release of nucleosomes from the complexes. This inhibition is preserved even at the high concentration of NAD^+^ (100 μM). Similarity to the E_PR_ profiles of the nucleosome-PARP1 in the presence and absence of NAD^+^ indicates that PARP1 is trapped in the complex and continues to maintain the altered structure of nucleosomal DNA. EMSA confirms that the addition of NAD+ does not release nucleosomes from the nucleosome-PARP1 complexes at the 10 µM concentration of Tala or Ola (Figure 3b,d). In contrast, Veli (10 μM) retains PARP1 in complexes with nucleosomes weaker than Ola and Tala: a significant proportion of nucleosomes dissociates from the complexes with PARP1 in the presence of 100 μM NAD+ and their intact structure is restored, as evidenced by the appearance of a shoulder in the E_PR_ profile in the 0.65–1.0 range (Figure 3e).

Differences between inhibitors in their ability to trap PARP1 becomes more evident when PARPi concentrations decrease, while the concentration of NAD+ is maintained constant (100 μM). Tala traps PARP1 in complexes with nucleosomes at a concentration higher than 0.1 μM (Figure 3c,d). Ola traps PARP1 at a concentration higher than 1 μM (Figure 3a,b). Veli partially traps PARP1 in the complexes at 10 μM, but cannot compete with NAD+ (100 µM) at a concentration of 1 µM and less (Figure 3e,f). In all cases, nucleosomes adopt the intact structure after a dissociation from nucleosome-PARP1-PARPi complexes, as evidenced by the shape of their E_PR_ profiles (Figure 3a,c,e).

## 4. Discussion

The PARPi-induced trapping of PARP1 at chromatin was clearly identified in the cells and was demonstrated to be the critical factor for PARPi cytotoxicity [11,12,14,15,16]. Since chromatin is a complex hierarchically organized system for the storage of DNA, most of which is packed into nucleosomes, the question arises about where the sites of PARP1 trapping localize. PARP1 can bind to both histone-free and nucleosomal DNA [37]. Previously published experiments in vitro show that PARP1 trapping occurs on linear DNA, although mechanisms of this trapping are debated [12,15,16,17,18,21]. Our data demonstrate that PARP1 trapping also occurs on nucleosomal DNA. Our data suggest that PARP1 trapping on nucleosomes occurs due to both a PARPi-mediated enhancement of PARP1 binding to nucleosomal DNA and the inhibition of PARylation; the data are in agreement with two previously proposed mechanisms of PARP1 trapping [11,12,14,15,16,17,18].

Tala, Ola, and Veli were found to differ considerably in their ability to enhance PARP1 binding to nucleosomal DNA. This enhancement is most pronounced for Tala, noticeably weaker for Ola, and negligible for Veli. It is possible that the binding of Ola or Tala to the catalytic center of PARP1 in the complex with a nucleosome causes conformational changes in PARP1, which increase the stability of the ternary complex, compared with the binary complex. Such structural changes were proposed in the reverse allosteric hypothesis as a reason of the increased affinity of PARP1 to DNA in the presence of PARPi [11,17,18], but were not found in the experiments with linear (free) DNA [12,15,16]. Such a structural adjustment of PARP1 on a nucleosome can occur because of the topological features of nucleosomal DNA, such as helix bending and closely spaced DNA gyres, as well as the interaction of PARP1 with histones, in particular, with tails of histones H3 or H4 [38,39,40]. The differences in the enhancement of PARP1 binding to nucleosomes in the presence of different PARPi could be induced by the differences in the network of interactions realized between PARPi and amino acid residues of PARP1 [15,41,42].

Considering the enhancement of PARP1 binding, the interactions between PARP1, nucleosomes, and PARPi can be described by a scheme

*K*_*a*1_           *K*_*a*2_ PARP1 + N + PARPi ⇄ PARP1:N + PARPi ⇄ PARPi:PARP1:N,(1)

where N is the nucleosome, *K*_a1_ and *K*_a_ are the association constants of the corresponding complexes, and *K*_*a*2_ > *K*_*a*1_. According to this scheme, the formation of ternary complexes shifts an equilibrium of the first reaction to the binary complex formation. An excess of PARPi will promote a transition of all the binary complexes to ternary complexes, thus provoking a complete recruitment of free PARP1 into complexes.

Affinities of Tala, Ola, and Veli to activated PARP1 are the basis of their ability to inhibit the catalytic activity of PARP1 and a factor affecting PARP1 trapping. A higher affinity of PARPi provides a more efficient competition with NAD+ for the binding to the enzyme and the inhibition of PARylation, including autoPARylation, that is required for the release of PARP1 from the complex with a nucleosome. Recently refined affinities of Tala, Ola, and Veli to activated PARP1 are 0.012, 0.97, and 0.96 nM [24].

From the comparison of PARPi affinities and the observed trapping of PARP1 at the nucleosomes, we can conclude that the relative affinities cannot fully explain the relative efficiencies of PARP1 trapping by the PARPi. Our data show that a higher PARP1 trapping by Ola as compared to Veli can result from the Ola-induced enhancement of PARP1 binding to a nucleosome. In the case of Tala, both a higher affinity and higher enhancement of PARP1 binding to a nucleosome provides the highest PARP1 trapping as compared to Ola and Veli (Figure 3). spFRET data show that the PARPi do not affect the conformation of nucleosomal DNA either in free nucleosomes or in the PARP1-nucleosome complexes that allows one to exclude an influence of this factor on the PARP1 trapping. Finally, relative PARP1 trapping efficiencies (Tala>Ola>Veli) observed at nucleosomes qualitatively reproduce the PARP1 trapping profile revealed for Tala, Ola, and Veli in cellular chromatin [11,21]. The data suggest that PARP1 trapping by PARPi in chromatin is implemented to a large extent at the level of nucleosomes.

According to spFRET analysis, nucleosomes released from the PARPi- PARP1-nucleosome complexes preserve an intact structure (Figure 3). Since the conformation of nucleosomal DNA is known to change considerably in sub-nucleosome particles missing any histones [43,44], the data suggest that the temporary PARP1 trapping in complex with a nucleosome as well as the processes of PARylation and PARP1 dissociation, are not accompanied by the loss of core histones.

In summary, our data suggest the following mechanism of increased cytotoxicity of PARPi, which are capable to trap PARP1 in complexes with DNA. The inhibitor-induced enhancement of PARP1 binding to nucleosomal DNA (as in the case of Ola and Tala) would increase the time that PARP1 spends in complex with DNA and decrease the free diffusion time of PARP1 in the nucleoplasm. It was proposed that PARP1 can move along DNA using a so-called “monkey-bar” mode [15,16,45]. By moving along DNA, the PARP1 will find DNA breaks faster than by 3D diffusion in nucleoplasm. Due to the increased affinity of PARP1 for damaged DNA [46,47,48], the PARP1 will delay at the sites of the DNA breaks and, in the presence of PARPi, it would block the repair for a long time because of the PARP1 inhibition by PARPi and interfere with the assembly of repair complexes that requires PARylation. A failure to repair DNA breaks is known to result in cell death that is successfully used in anticancer therapy.

## Data Availability

The data presented in this study are available on request from the corresponding authors. The data are not publicly available due to local regulations.
